# TRIM28 inhibits alternative lengthening of telomere phenotypes by protecting SETDB1 from degradation

**DOI:** 10.1186/s13578-021-00660-y

**Published:** 2021-07-30

**Authors:** Chuanle Wang, Zhou Songyang, Yan Huang

**Affiliations:** 1 MOE Key Laboratory of Gene Function and Regulation, Guangzhou Key Laboratory of Healthy Aging Research and SYSU-BCM Joint Research Center, School of Life Sciences, Sun Yat-Sen University, Guangzhou, 510275 China; 2 Sun Yat-Sen Memorial Hospital, Sun Yat-Sen University, Guangzhou, 510120 China; 3 Verna and Marrs Mclean Department of Biochemistry and Molecular Biology, Baylor College of Medicine, One Baylor Plaza, Houston, TX 77030 USA; 4 Bioland Laboratory (Guangzhou Regenerative Medicine and Health Guangdong Laboratory), Guangzhou, 510005 China

**Keywords:** TRIM28/KAP1, SETDB1, Alternative lengthening of telomere phenotypes, Heterochromatin

## Abstract

**Background:**

About 10–15% of tumor cells extend telomeres through the alternative lengthening of telomeres (ALT) mechanism, which is a recombination-dependent replication pathway. It is generally believed that ALT cells are related to the chromatin modification of telomeres. However, the mechanism of ALT needs to be further explored.

**Results:**

Here we found that TRIM28/KAP1 is preferentially located on the telomeres of ALT cells and interacts with telomeric shelterin/telosome complex. Knocking down TRIM28 in ALT cells delayed cell growth, decreased the level of C-circle which is one kind of extrachromosomal circular telomeric DNA, increased the frequency of ALT-associated promyelocytic leukemia bodies (APBs), led to telomere prolongation and increased the telomere sister chromatid exchange in ALT cells. Mechanistically, TRIM28 protects telomere histone methyltransferase SETDB1 from degradation, thus maintaining the H3K9me3 heterochromatin state of telomere DNA.

**Conclusions:**

Our work provides a model that TRIM28 inhibits alternative lengthening of telomere phenotypes by protecting SETDB1 from degradation. In general, our results reveal the mechanism of telomere heterochromatin maintenance and its effect on ALT, and TRIM28 may serve as a target for the treatment of ALT tumor cells.

**Supplementary Information:**

The online version contains supplementary material available at 10.1186/s13578-021-00660-y.

## Introduction

Telomeres are special structures at the ends of eukaryotic chromosomes, which is composed of TTAGGG repeat sequence and a protein complex called "telosome" or "shelterin" [1, 2]. Telomeres protect the integrity and stability of chromosomes and avoid chromosome terminal fusion and DNA damage response [3]. Due to end-replication problem, telomeres of human normal somatic cells would gradually erode with cell division [4], when telomeres shorten to a certain extent, cells will stop dividing and enter a state of senescence, so telomeres are called the "life clock" [5–7]. Telomere dysfunction would induce multiple human aging related diseases including dyskeratosis congenita (DC), idiopathic pulmonary fibrosis (IPF), and cancer [8–10].

On the other hand, tumor cells are able to break through "Hayflick limit", i.e., to achieve cell immortalization. 85–90% of tumor cells extend telomeres by activating telomerase, while 10–15% of them utilize telomerase-independent mechanism called the alternative lengthening of telomeres (ALT) [11]. ALT is a homologous recombination (HR)-directed telomere synthesis pathway [12]. There are obvious differences in telomere length between ALT cells and telomerase positive cells, which may be caused by the rapid deletion or lengthening of telomeres. The chromosomal telomere length is highly heterogeneous [13]. In addition, the extra-chromosomal telomere fragments, including C-circles and C-overhangs, are released in ALT cells due to the collapse of replication forks [14]. Another remarkable feature of ALT is the existence of ALT-associated promyelocytic leukemia bodies (APBs). APBs function as nuclear structures in which telomere DNA replication and recombination occurs spatiotemporally. In ALT cells, each APB contains on average 2–5 telomeres [15, 16].

The mutation or loss of α-thalassemia/mental retardation syndrome, X-linked (ATRX) and death domain-associated protein (DAXX) seems to be the key determinant of the emergence and maintenance of ALT [17]. According to previous studies, ATRX protein was low or absent in 86% of human ALT cell lines [18]. Re-introducing wild-type ATRX in ALT cells inhibits the ALT activity of tumor cells [19]. ATRX and DAXX form a chromatin remodeling complex, which is responsible for the deposition of histone H3.3 on telomere [20]. In ATRX/DAXX mutant ALT cells, histone H3.3 is not assembled correctly to achieve chromatin compression [21]. The unique epigenetic status of telomere, including histone or telomere DNA modification caused by ATRX/DAXX mutation and the telomeric nucleosome density provide chromatin adaptability for HR-mediated ALT pathway.

Initial studies suggested that RAD51-mediated recombination is the basis for the occurrence of ALT [22, 23]. Telomeric repeat-containing RNA (TERRA) forms R-Loop structure on telomere and triggers telomere fragility. RAD51 participates in the formation of R-loop and promotes homologous recombination [24]. However, several studies believed that RAD51 is not necessary for break-induced telomere synthesis (BITS) mediated ALT [25–27]. At present, the break-induced replication (BIR) is considered to be the basis of the ALT pathway and is independent of RAD51 [25].

Telomere belongs to heterochromatin region and enriches many heterochromatin-related epigenetic proteins. ALT is corelated to the epigenetic modification state of telomere DNA, which is also one of the most important mechanisms regulating the structural stability of telomere. ATRX interacts with DNA methyltransferase 1 (DNMT1), and the deletion of ATRX alters the levels of DNA methylation in subtelomere regions [28]. Previously we found hetrochromatin protein 1 binding protein 3 (HP1BP3) increases the level of H3K9me3 modification on telomere and stabilizes the compacted structure of telomere chromatin to inhibit ALT phenotypes and ALT tumor cell growth [29]. However, a few studies have suggested that the ALT pathway is promoted by heterochromatin formation, Gauchier et al. identified telomere-associated proteins in mouse embryonic stem cells by proteomics of isolated chromatin segments (PICh) [30]. They showed that SETDB1 is located on telomeres, while SUV39H is mainly enriched on pericentromeres. The deletion of SETDB1 decreases the level of heterochromatin marker H3K9me3 on telomere and the frequency of APBs, while the deletion of SUV39H increases the telomeric H3K9me3 level [31]. Their findings are inconsistent with previous reports [32]. These controversy results indicate that the ALT process is sophisticated and needs to be explored more precisely.

TRIM28 or KAP1, is a member of the tripartite motif family, has received extensive attention since it was discovered in 1996 [33, 34]. It was first described as a repressor member of the Krüppel-associated Box zinc finger protein (KRAB-ZFP) family [34]. With deepening exploration, TRIM28 has been found to be involved in a wide range of biological processes in cells. For example, it facilitates the formation of heterochromatin, mediates DNA damage response, stimulates epithelial-mesenchymal transition (EMT) and helps to maintain the pluripotency of stem cells. Sumoylation of TRIM28 mediates gene silencing by recruiting H3K9-specific histone methyltransferase SETDB1 and nucleosome remodeling and deacetylation (NuRD) complex [35]. TRIM28 helps to maintain the pluripotent state of mouse embryonic stem cells (mESCs), and the absence of TRIM28 significantly down-regulates the pluripotency markers Oct4, Sox2 and Nanog, thus induces stem cells to differentiate into primitive ectoderm lineage [36]. During early mouse embryonic development, E3 ubiquitin ligase Dcaf11 targets TRIM28 and promotes its degradation, thus relieving the transcriptional inhibition of Zscan4 and activating the ALT pathway of mESCs [37]. Endogenous retroviruses pose a great threat to the stability of the genome. TRIM28 complex facilitates to silence the transcription of retroviruses, and the deletion of TRIM28 leads to a series of up-regulation of endogenous retroviruses (ERVs) in mESCs [38]. Consistently, TRIM28 loss of function upregulates ERVs and facilitates the induced pluripotent stem cell (iPSCs) reprogramming [39]. The SUMO modification of CDK9 residues by TRIM28 prevented the interaction between CDK9 and Cyclin T1 and effectively inhibited the expression of HIV-1 [40]. Upon DNA double-strand breaks, ATM and/or ATR-dependent DNA damage response pathways are activated. Phosphorylatation of TRIM28 by ATM interferes with its binding to chromatin remodeling factors, increasing the accessibility of chromatin to DNA repair proteins [41].

TRIM28 is highly expressed in many malignant tumors, its high expression in ovarian cancer and cervical cancer promotes tumor invasion and metastasis [42, 43]. The interaction between TRIM28 and TRIM24 protects TRIM24 from SPOP-mediated ubiquitin degradation and promotes the progression of prostate cancer [44]. TRIM28 also regulates the epithelial-mesenchymal transition (EMT) pathway and enable tumor cells to acquire mesenchymal phenotype and become more aggressive [45].

Telomere associated proteins play an important role in maintaining telomere integrity. Although multiple progress has been made in the study of telomere associated proteins, the identification of new telomere associated proteins is of great significance for understanding telomere regulation. In 2009, researchers developed a method called proteomics of isolated chromatin segments (PICh) [30], in which they used telomere probes to pull-down and identify by mass spectrometry telomere-associated proteins. Many new telomere-associated proteins were identified and studied later on. For example, BUB3 promotes telomere chromosome replication, and HMBOX1 participates in telomere maintenance in ALT cells [48, 49]. TRIM28 was also identified as telomere-associated proteins in both telomerase positive HeLa and ALT-mediated Wi38-VA13 cells [30]. In 2017, another group also found that TRIM28 locates on telomeres of many species by systematic phylointeractomics screening, including human and mouse, suggesting the evolutionarily conserved role of TRIM28 on telomere [50]. However, the precise role of TRIM28 on telomere maintenance remained largely unknown. In this study, we briefly demonstrated the function and mechanism of TRIM28 in the ALT-mediated telomere maintenance. Our findings provide new insights into the occurrence of ALT and may provide potential targets for the treatment of ALT tumor cells.

## Materials and methods

### Cell lines and antibodies

The U2OS, Wi38-VA13 and HEK293T cells were cultured in dulbecco's modified eagle medium (DMEM) containing 10% fetal bovine serum (FBS) and cultured in an incubator containing 5% CO2 at 37 ℃. Human full-length TRIM28 cDNA was cloned into pDEST27 (Invitrogen) with GST tag and Plenti-HAFL-puro with HA-Flag (HAFL) double tags.

Cells were infected with lentivirus corresponding to shRNAs or overexpression vectors and selected with puromycin. Sense and antisense DNA oligos designed according to siRNA sequences were synthesized and cloned into pLKO.1-GFP vector. The siRNA sequences are: siTRIM28-1, 5′-CCUggCUCUgUUCUCUgUCCU-3′; siTRIM28-2, 5′-CUgAgACCAAACCUgUgCUUA-3′; siLuci, 5′-CUUACGCUGAGUACUUCGA -3′.

Antibodies used in this study include: rabbit polyclonal anti-TRIM28 (Abcam), rabbit polyclonal anti-Flag (Abmart), mouse monoclonal anti-tubulin (Sigma), rabbit polyclonal anti-GAPDH (Abmart), rabbit polyclonal anti-53BP1 (Novus), rabbit polyclonal anti-GST (Abmart), mouse monoclonal anti-PML (Millipore), rabbit polyclonal anti-Histone H3 (Abcam), rabbit polyclonal anti-H3K9me3 (Abcam) and rabbit polyclonal anti-SETDB1 (Proteintech).

### C-circles (CC) assay

C-circle assay was performed as described previously [46]. 250 ng genomic DNA was digested with 0.25 µl Hinf1 and 0.25 µl Rsa1 (4 U/µg) at 37 °C for 4 h, diluted to the designed concentration (25, 50, or 100 ng of DNA per 10 µl volume) and combined in a 10 µl of reaction mixture (5 µg BSA, 1 mM dATP, 1 mM dTTP, 1 mM dGTP, Ф29 buffer, and 5 U Ф29 DNA polymerase (NEB). The reaction system was incubated at 30 °C for 8 h in a PCR instrument, then inactivated at 65 °C for 20 min. The remaining sample was diluted to 20 μl with 2 × SSC, denatured in 95 °C for 10 min and quickly cooled down to 4 °C, as the sample for internal reference. 10 μl of each sample was used for southern blotting. The amplified C-circle samples were hybridized with C-rich telomere probe, and the samples for internal reference were hybridized with Alu probe. TeloC probe: 5′-Biotin-CCCTAACCCTAACCCTAA-3′; Alu probe: 5′-Biotin-GGCCGGGCGCGGTGGCTCACGCCTGTAATCCCAGCA-3′.

### Telomere DNA-FISH

DNA-FISH was performed as previously described [47]. Cells were fixed 30 min on ice with 4% paraformaldehyde. After washing with 1 × PBS for 3 times, the glass slides were dehydrated with 70%, 90% and 100% ethanol respectively and dried under the condition of RT for 15 min. The FITC-labeled (CCCTAA)_3_ probe (0.5 μg/ml) (Panagene, Korea) was added to the samples. After denaturing 5 min at 80 °C, slides were incubated at 37 °C in wet chamber for 2 h. Then the slides were washed with washing solution (70% formamide, 10 mM Tris pH 7.4) for 3 times. Finally, the slides were stained with DAPI and detected by fluorescence microscope. The fluorescence intensity was quantified by ImageJ and Student's t-test was calculated in statistics.

### Chromosome orientation fluorescent insitu hybridization (CO-FISH)

Cells were subcultured in 5′-bromo-2′-deoxyuridine (BrdU, 10 μM; Sigma) for 14 h and then cultured with Nocodazol (0.5 μg/ml) for another 6 h. Then cells were digested and treated with 0.075 M KCl hypotonic solution, fixed and dropped on slides, CO-FISH was performed using a Cy3-labeled (TTAGGG)_3_ probe and a FITC-labeled (CCCTAA)_3_ probe as described previously [47].

### GST Pull-down assay

Cell samples were lysed with NETN lysis buffer for 30 min and oscillated every 10 min. Samples were centrifuged at 12,000 rpm at 4 ℃ for 10 min. 20 μl supernatants were reserved as input. The rest of the samples were transfered into a new tube, added with 20 μl precleared GST beads and rotated for over 4 h. The samples were washed with 400 μl precooled NETN lysis for three times. 20 μl NETN lysate and 5 μl protein loading buffer (5×) were added to each tube as IP sample. The input and IP samples were analyzed with 10% SDS-PAGE gel and detected by western blot.

### immunofluorescence-fluorescent in situ hybridization (IF-FISH)

Cells of about 70% density on the slide was washed with PBS for 3 times, fixed with 4% paraformaldehyde (PFA) for 30 min and treated with appropriate amount of permeabilization solution for 30 min. The slides were blocked with 5% goat serum at room temperature for 1 h, incubated with the primary antibody at 4 ℃ overnight or at room temperature for 2 h, washed with PBS for 3 times, and incubated with the second antibody at room temperature for 1 h. The slides were refixed with 4% PFA for 30 min, dehydrated with 70%, 90% and 100% ethanol, incubated with fluorescent-labeled telomere probe at 85 ℃ for 5 min and then 37 ℃ overnight. After washed twice, the slides were dried and stained with DAPI.

## Results

### TRIM28 is preferentially located on the telomere of ALT cells and interacts with telosome/shelterin complex

**Fig. 1:**
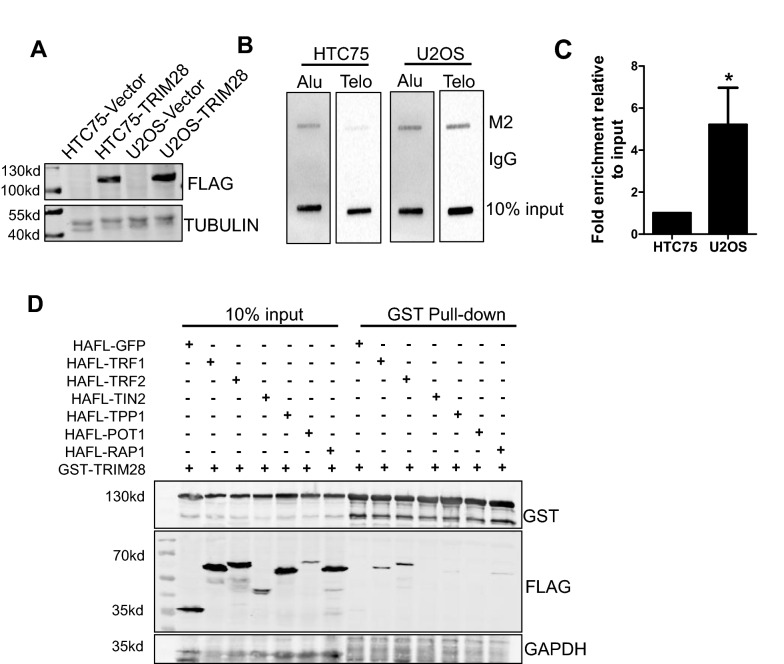
TRIM28 is preferentially located on the telomere of ALT cells and interacts with shelterin/telosome complex. **A** Lysates of HTC75 and U2OS cells stably expressing HAFL-TRIM28 were detected by western blot. **B** TRIM28 over-expressed cell lines were used in telomere ChIP assays with M2 beads, the precipitated DNA was slot-blotted and probed with a biotinylated telomere probe. IgG served as a negative control. **C** The data of (**B**) was repeated twice by independent experiments, then quantified and plotted as a histogram. *P < 0.05, Student’s t-test. **D** Detection of the interaction between TRIM28 and shelterin complex subunits by GST pull-down assay in 293T

In order to explore whether TRIM28 is exactly located on telomere and whether its telomere localization has cellular specificity, we overexpressed HA-FLAG tagged TRIM28 in telomerase positive HTC75 cells and ALT-dependent U2OS cells respectively (Fig. 1A). Telomere chromatin immunoprecipitation (ChIP) assays showed that TRIM28 is located on telomeres, and TRIM28 is preferentially located on telomeres of ALT -dependent U2OS cells compared to telomerase positive HTC75 cells (Fig. 1B, C).

We wondered how TRIM28 is recruited to telomere. Telomere is bond by telosome/shelterin complex; thus, we performed the GST pul-down assay. Plasmids that express TRIM28 with GST tag and telosome/shelterin subunits with HA-Flag double tags were co-transfected into HEK293T cells. The result showed that TRIM28 interacts with TRF1, TRF2 (Fig. 1D). At the same time, we performed bimolecular fluorescent complimentary (BiFC) experiment. Two proteins with potential interaction are fused with the N-terminal and C-terminal of GFP respectively. When the two candidate proteins bind to each other, the N-terminal and C-terminal of GFP protein will be close to each other and emit fluorescence, which can be detected by flow cytometry and microscopy (Additional file 1: Fig. S1A) [51]. BiFC results showed that TRIM28 interacts with shelterin subunits (Additional file 1: Fig. S1B, C). The interaction of TRIM28 with shelterin components indicates that TRIM28 may localize to telomere via its association with shelterin subunits.

### TRIM28 knockdown increases telomere dysfunction-induced foci

**Fig. 2:**
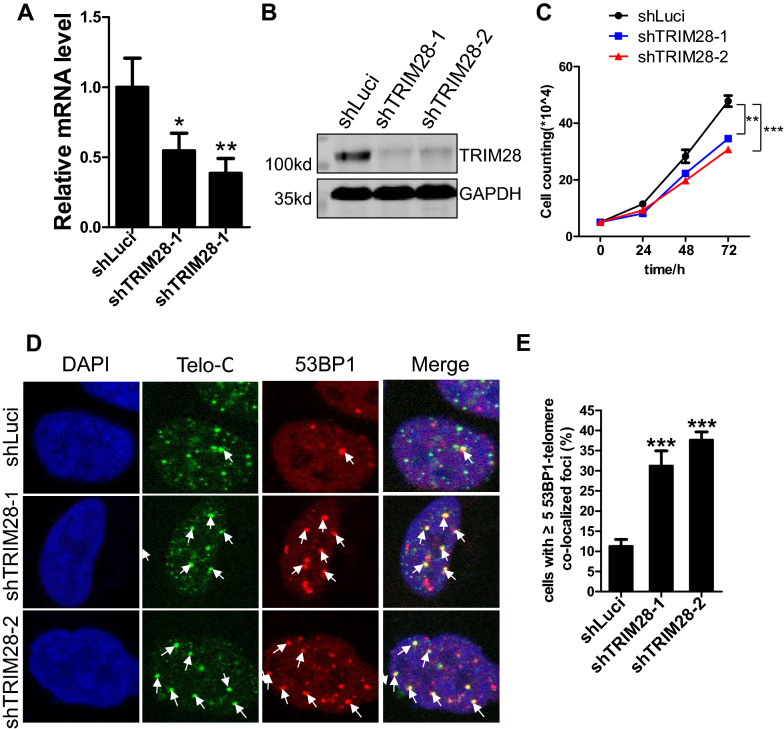
TRIM28 knockdown induces telomere dysfunction-induced foci. Knocking-down efficiency of shRNAs targeting TRIM28 were detected by Q-PCR (**A**) and by western blot (**B**). *P < 0.05, **P < 0.01, Student’s t-test. **C** The effect of TRIM28 deletion on the growth of U2OS cells was detected by cell counting. The initial cell number was 50,000. **P < 0.01, ***P < 0.001, Student’s t-test. **D** U2OS cells infected with viruses of control (shLuci) or two different shRNAs against TRIM28 were stained by IF-FISH assay with a telomere probe (green) and anti-53BP1 antibodies (red). DAPI was used to show the nuclei. Arrows indicate colocalization signals. **E** The data of (**D**) was quantified and plotted as a histogram, about 200 cells were analyzed for each line and those with ≥ 5 53BP1-telomere co-localized foci were counted as positive. Error bars represent SD (n = 3). ***P < 0.001, Student’s t-test

To gain insight into the function of TRIM28 in ALT cells, we designed and cloned two shRNAs in vectors with GFP fluorescence to knock down TRIM28 in U2OS cells (Additional file 2: Fig. S2A). Q-PCR and western blot results showed that the two shRNAs efficiently reduce the mRNA and protein level of TRIM28 in U2OS (Fig. 2A, B). After infecting U2OS cells with shRNA lentivirus for 36 h, the fluorescence signal appears in the cells under the fluorescence microscope (Additional file 2: Fig. S2A). Compared with the negative control, the growth of TRIM28-deficient U2OS cells slowed down (Fig. 2C). We then detected the telomeric DNA damage response after knocking down TRIM28 in U2OS cells. 53BP1 represents the DNA damage response and its colocalization with telomere indicates telomere-specific DNA damage foci called telomere dysfunction-induced foci (TIF) [52]. The results showed that TRIM28 knockdown increases TIF signals significantly (Fig. 2D, E).

### TRIM28 knockdown reduces the C-circle level of ALT cells

**Fig. 3:**
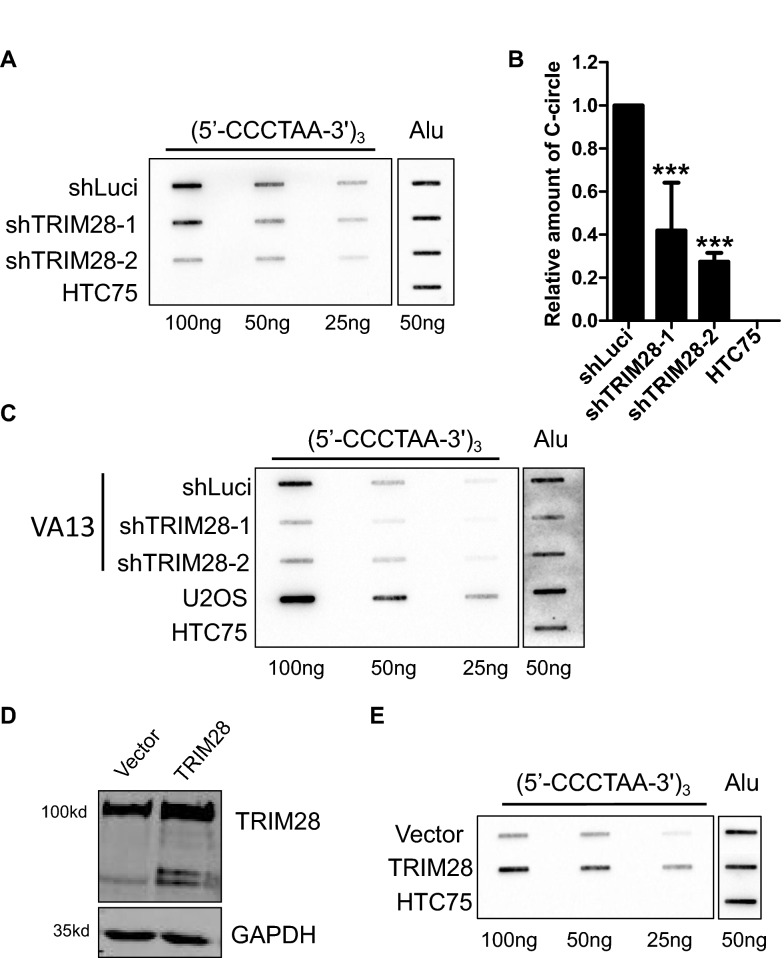
TRIM28 knockdown decreases the C-circle level of U2OS cells. **A** U2OS Genomic DNA (25, 50, or 100 ng) from shLuci and shTRIM28 cells were used for the C-circle assay. An Alu repeat probe served as internal control. **B** Quantification histogram of data from (**A**). Intensity values from TRIM28 knockdown cells were normalized to the control cells. Error bars indicate standard error (n = 3). ***P < 0.001, Student’s t-test. **C** C-circle formation assays via ∅29 amplification and slot blotting in VA13 cells. **D** Overexpression of TRIM28 in U2OS cells was detected with TRIM28 antibodies. **E** C-circle formation assays via ∅29 amplification and slot blotting in TRIM28 overexpressed U2OS cells

C-circle is one of the multiple biomarkers of ALT cells [46]. Some studies have suggested that the production of C-circle is related to telomere DNA damage, especially double strand breaks [53]. We then detected the C-circle levels in U2OS cells after TRIM28 deletion, and found that TRIM28 deletion significantly reduces the C-circle levels (Fig. 3A, B). Similar data was observed in another ALT cell line Wi38-VA13 (Fig. 3C). Consistently, overexpression of TRIM28 significantly increased the level of C-circle in U2OS cells (Fig. 3D, E).

### TRIM28 deletion promotes APB formation and telomere lengthening

**Fig. 4:**
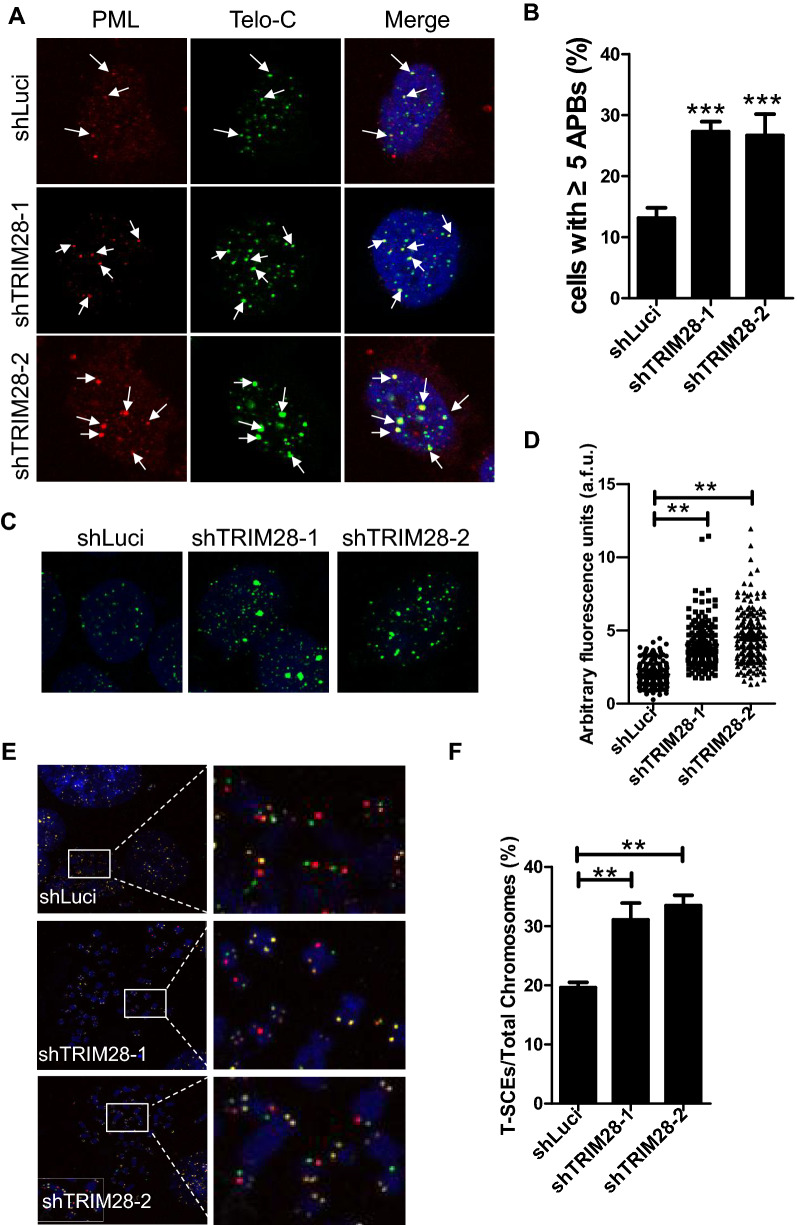
TRIM28 deletion promotes APB formation and telomere extension. **A** IF-FISH of TRIM28 KD U2OS cells was performed using a telomere probe (green) and antibodies against PML (red). DAPI was used to stain nuclei. Arrows indicate colocalization signals. **B** Results from (**A**) were quantified. About 200 cells were observed and those with ≥ 5 APBs were counted as positive. Error bars indicate standard error (n = 3). ***P < 0.001, Student’s t-test. **C** Telomere length of U2OS cells was measured by telomere DNA-FISH after TRIM28 knockout. DAPI was used to stain chromosomes. **D** Data from (**C**) were quantified to assess relative telomere length, more than 150 cells were observed for each cell line. **P < 0.01, Student’s t-test. **E** T-SCEs in TRIM28-depleted U2OS cells were shown. Briefly, after the chromosome was fixed and digested by exonuclease III, Cy3-labeled (TTAGGG)_3_ (red signal) and FITC-labeled (CCCTAA)_3_ (green signal) fluorescent probes were used for in situ hybridization, the single strand region showed a single fluorescent signal, and the recombination region showed yellow signal due to the existence of double strands. The enlarged image shown the typical T-SCEs. **F** Quantification of (**E**). More than 200 chromosomes were examined. **P < 0.01, Student’s t-test

APB is a special nuclear body that exists only in ALT cells. As the active center of ALT, APB includes telomere DNA and its associated shelterin complex [15]. We found that interfering with the expression of TRIM28 significantly increases the level of APBs, indicating a higher level of telomere homologous recombination (HR) after TRIM28 depletion (Fig. 4A, B). Thus, we analyzed the telomere length of U2OS after TRIM28 deletion. Both DNA-FISH and Q-FISH results showed that telomere lengthens significantly after TRIM28 deletion (Fig. 4C, D, Additional file 3: Fig. S3). High frequency of telomere sister chromatid exchange (T-SCE) is another important biological marker of ALT cells. We found that TRIM28 deletion leads to a significant increase in T-SCE frequency in U2OS cells (Fig. 4E, F). These results indicate TRIM28 depletion may promote ALT phenotypes in U2OS cells.

### TRIM28 promotes H3K9me3 occupancy on telomere in ALT cells

**Fig. 5:**
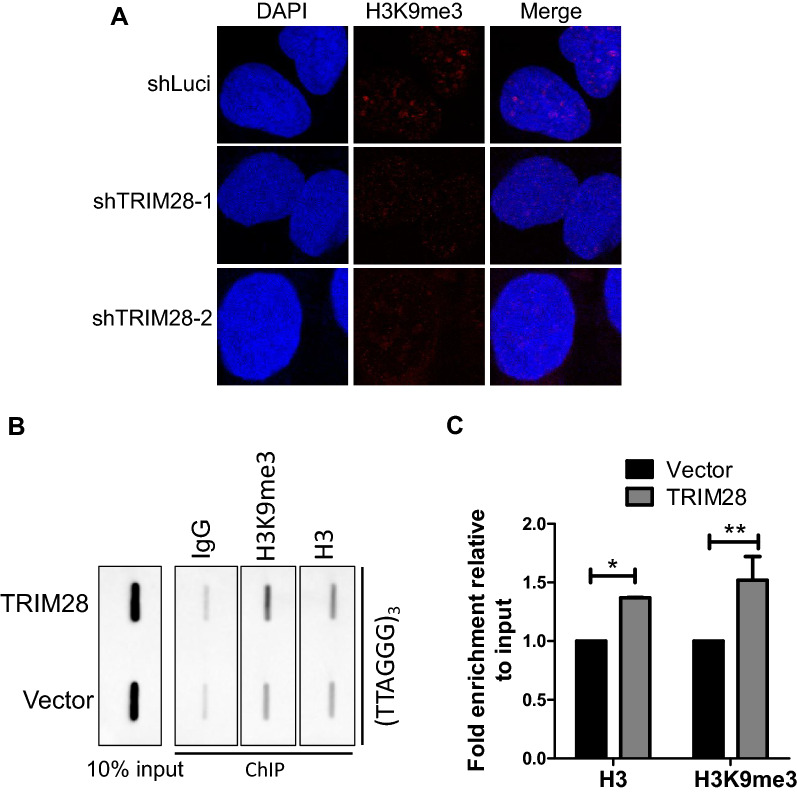
TRIM28 promotes H3K9me3 occupancy on telomere in ALT cells. **A** IF assay of TRIM28-deficient U2OS cells was performed using antibodies against H3K9me3. **B** Telomere ChIP analysis in U2OS cells with overexpressed TRIM28 or vector were performed using the indicated antibodies. Rabbit IgG served as a negative control. The precipitated DNA was slot blotted and probed with biotin-labeled telomere and Alu probes. **C** Relative signals from (**B**) were quantified and normalized to histone H3 signals. *P < 0.05, **P < 0.01, Student’s t-test

TRIM28 is widely involved in the formation of heterochromatin [34, 54, 55]. The IF result showed that histone H3K9me3 modification decreases significantly after TRIM28 knockdown (Fig. 5A), indicating TRIM28 is required for maintaining heterochromatin H3K9me3 levels. By telomere chromatin immunoprecipitation (ChIP), we detected H3K9me3 modification at telomere in TRIM28 overexpressed U2OS cell lines and found that TRIM28 promotes H3K9me3 modification enriched at telomeres (Fig. 5B, C).

### TRIM28 promotes the protein stablity of SETDB1

**Fig. 6:**
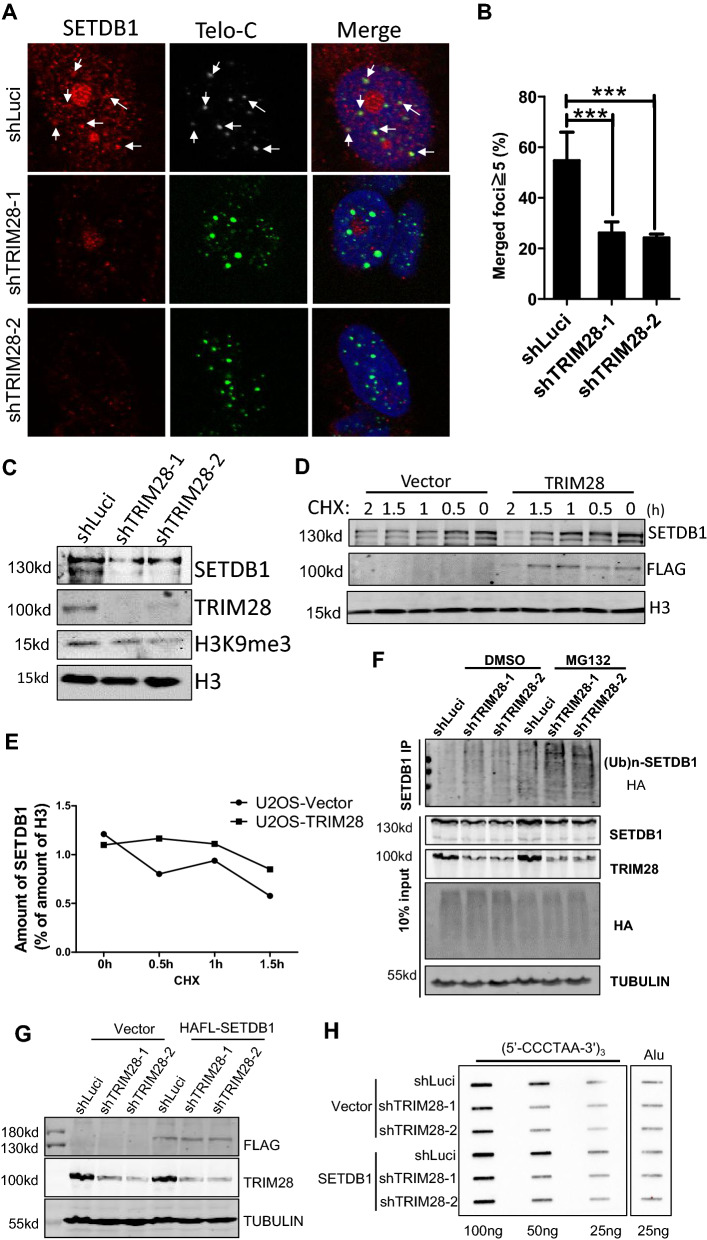
TRIM28 positively regulates the protein stablity of SETDB1. **A** IF-FISH of TRIM28 KD U2OS cells was performed using a telomere probe (green) and antibodies against SETDB1 (red). Arrows indicate co-stained signals. **B** 200 cells were analyzed for each line and those with ≥ 5 SETDB1-telomere co-localized foci were counted as positive. Error bars represent SD (n = 3). ***P < 0.001, Student’s t-test. **C** Cells from (**A**) were lysed for western blot analysis with the indicated antibodies. **D** TRIM28 overexpressing U2OS cells were treated with cycloheximide for indicated timespan and the protein level of SETDB1 was detected by western blot. **E** The signal of (**D**) were quantified. **F** Cells from (**A**) were transfected with HA-Ub, treated with DMSO or MG132 for 4 h, then the ubiquitin level of SETDB1 was detected by western blot. **G** TRIM28-deficient U2OS cell lines were overexpressed with Vector control or SETDB1 and cell lysates were detected by western blot with the indicated antibodies. **H** Genomic DNA from cell lines (**G**) were used for the C-circle assay

Histone methyltransferase SETDB1 catalyzes the trimethylation of histone H3 and maintain the transcriptional siliencing of chromatin. Interestingly, SETDB1 interacts with TRIM28 to mediate retrovirus silencing [55–57]. We found that upon the deletion of TRIM28 in U2OS cells, SETDB1 levels, both in total and at telomeres, are significantly decreased (Fig. 6A, B), accompanied by a decrease in SETDB1 protein level as well as the histone modification H3K9me3 level (Fig. 6C). These results suggested that TRIM28 may be required to maintain the protein stability of SETDB1.

We treated the TRIM28-overexpressing U2OS cells with protein synthesis inhibitor cycloheximide (CHX) for different time period, and western blot results showed that overexpression of TRIM28 stabilizes the protein level of SETDB1 by delaying its degradation rate (Fig. 6D, E). Previous studies have shown that TRIM28 protects TRIM24 from SPOP-mediated ubiquitin degradation and promotes the progression of prostate cancer [44]. TRIM28 was also reported to stabilize and promote the accumulation of α-synuclein (α-Syn) and tau proteins in the nucleus of neurons, and promote the progression of neurodegenerative diseases [58]. We speculated that TRIM28 may also facilitate to maintain the protein stability of SETDB1 in a similar way. We transfected the ubiquitin plasmid HA-Ub in TRIM28-deleted U2OS cells. Expectedly, the ubiquitin level of SETDB1 increased after TRIM28 deletion (Fig. 6F). Furthermore, after 36 h of infection with two shRNA lentivirus targeting SETDB1 respectively, the cell morphology was changed, which is similar to that after deletion of TRIM28 (Additional file 4: Fig. S4A), and deletion of SETDB1 also decreased the level of C-circle in U2OS cells (Additional file 4: Fig. S4B). In order to elucidate the SETDB1-TRIM28 axis more precisely, we further overexpressed SETDB1 in TRIM28-deficient U2OS cell lines and found reintroduced SETDB1 seems to partially rescue the decreased level of C-circle in TRIM28-deficient cells (Fig. 6G, H). Collectively, our results showed that SETDB1 may function in phenocopy with TRIM28 and TRIM28 may stabilize SETDB1 on telomere.

## Discussion

TRIM28 was first described as nuclear corepressor for KRAB domain-containing zinc finger proteins (KRAB-ZFPs), which mediates gene silencing [33]. We found that TRIM28 interacts with telosome/shelterin subunits TRF1, TRF2 (Fig. 1D), suggesting that TRIM28 may play an important role in telomere maintenance. As TRIM28 was preferentially located on the telomere of ALT cells (Fig. 1B, C), we speculated TRIM28 may regulate telomere maintenance in ALT cells.

ALT is a telomere lengthening pathway mainly based on homologous recombination (HR), accompanied by extensive genome rearrangement and endogenous DNA damage. C-circle is one of the most significant biological characteristics of ALT tumor cells [46]. However, C-circles have also been found in cells under replication stress with no other ALT phenotypes [59]. Also, it was reported that the production of C-circle is related to the collapse of replication forks caused by excessive telomere replication stress [14]. In our study, TRIM28 deletion caused a decrease in the H3K9me3 level of telomere DNA (Fig. 5B, C), accompanied by the emergence of DNA damage response (Fig. 2D, E). Although the heterochromatin state of telomere is not conducive to telomere replication, the decrease of H3K9me3 in the whole cell cycle may accumulate more DNA damage response and eventually increase the DNA replication stress. Thus we speculated that the production of C-circle is the result of the crosstalk between replication stress and telomere chromatin state.

A recent study has found that Dcaf11 targets TRIM28 for ubiquitination-mediated degradation in mouse embryonic stem cells to remove heterochromatic H3K9me3 at telomere regions and activate the Zscan4-mediated ALT mechanism [37]. The negative roles of TRIM28 and telomeric H3K9me3 in ALT are consistent to our results. Zscan4 is only expressed in two-cell embryos and a small proportion of embryonic stem cells at a given time [60], suggesting that it may not be the target for TRIM28 to regulate the ALT pathway in cancer cells. Our studies suggest that TRIM28 may directly target SETDB1 and participate in the regulation process of ALT.

We found that TRIM28 stabilizes SETDB1 to maintain the modification level of telomere H3K9me3 (Fig. 6A–C). It has been reported that SETDB1-dependent telomere heterochromatin is the cause for promoting ALT, while SUV39H1 is not required for maintainence of telomere heterochromatin state [31]. The precise molecular mechanism of this difference is elusive. It is worth noting that TRIM28, as an E3 ligase that catalyzes SUMO2-PCNA conjugation and effectively resolves transcription-replication conflict (TRC). The deletion of TRIM28 will lead to the accumulation of RNAPII at the TRC sites and induce DNA damage [61]. Whether this process is involved in the activation of ALT pathway needs to be further explored. Although our BiFC data confirmed that TRIM28 interacts with TPP1 (Additional file 1: Fig. S1B, C), which is a shelterin subunit for recruiting telomerase, immunoprecipitation combined with telomerase activity assay (IP-TRAP) of TRIM28 found that TRIM28 does not interact with telomerase (data not shown), so it further confirmed the role of TRIM28 in ALT cells. Collectively, our work combined revailed a role of TRIM28 in telomere heterochromatin maintenance. TRIM28 may provide a potential therapeutic target for ALT tumors.

## Supplementary Information

**Additional file 1. **Bimolecular fluorescent complimentary (BIFC) of TRIM28 and shelterin subunits.

**Additional file 2. **Morphology of U2OS cells after knocking down TRIM28.

**Additional file 3. **TRIM28 deletion promotes telomere elongation.

**Additional file 4. **U2OS cells of knocking down SETDB1 show morphology and decreased C-circle levels similar to the TRIM28-deficient cells.

## Data Availability

The datasets used and/or analysed during the current study are available from the corresponding author on reasonable request.
