# Supplementary material for: TRIM28 inhibits alternative lengthening of telomere phenotypes by protecting SETDB1 from degradation
Source: Cell Biosci. 2021 Jul 30;11:149. doi: 10.1186/s13578-021-00660-y (PMC8325274; doi:10.1186/s13578-021-00660-y)
Supplement: Supplementary file 1 — Additional file 1. Bimolecular fluorescent complimentary (BIFC) of TRIM28 and shelterin subunits. [file 13578_2021_660_MOESM1_ESM.pdf]

Additional File 1: Figure S1

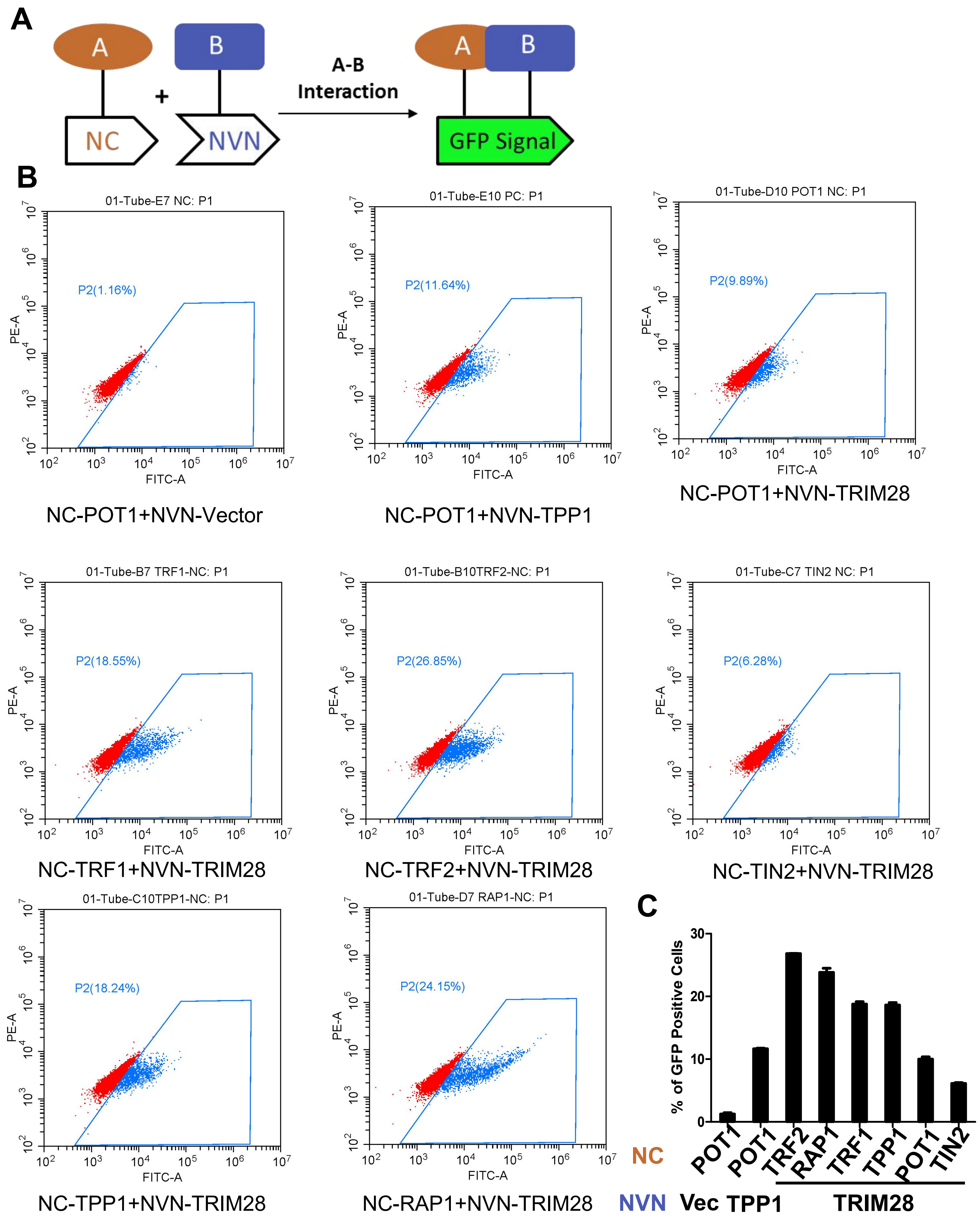

Figure S1. Bimolecular fluorescent complimentary (BIFC) of TRIM28 and shelterin subunits. (A) The schematic diagram of BiFC. (B) The BIFC results of TRIM28 and shelterin subunits were detected by flow cytometry, in which NC-POT1+NVN-Vector and NC-POT1+NVN-TPP1 used as the negative and positive control respectively. NVN refers to connecting the N-terminal of GFP to the N-terminal of the target protein, and NC refers to connecting the C-terminal of GFP to the N-terminal of the target protein. (C) The data of (B) is quantified and plotted as a histogram.
