# Supplementary material for: TRIM28 inhibits alternative lengthening of telomere phenotypes by protecting SETDB1 from degradation
Source: Cell Biosci. 2021 Jul 30;11:149. doi: 10.1186/s13578-021-00660-y (PMC8325274; doi:10.1186/s13578-021-00660-y)
Supplement: Supplementary file 2 — Additional file 2. Morphology of U2OS cells after knocking down TRIM28. [file 13578_2021_660_MOESM2_ESM.pdf]

Additional File 2: Figure S2

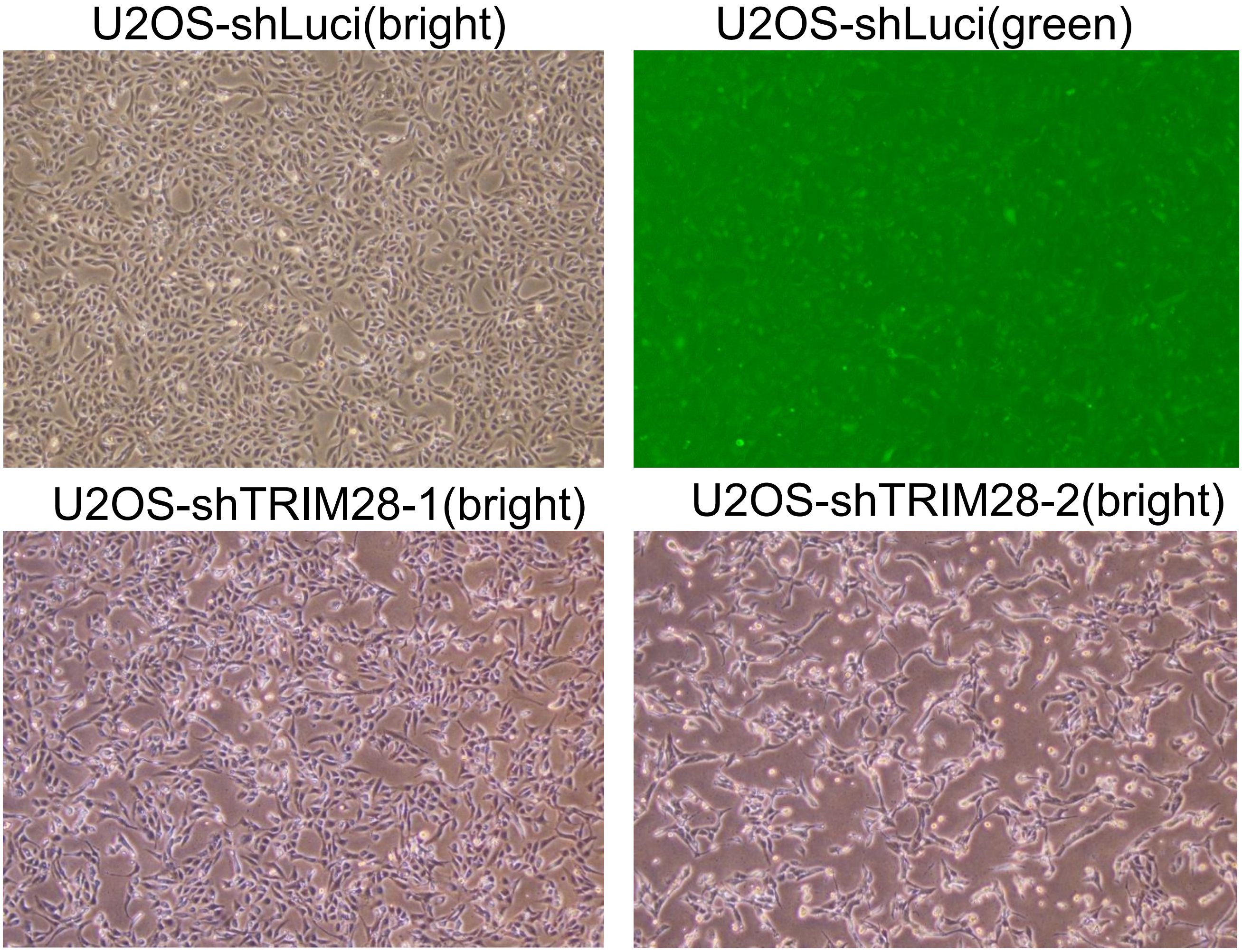

Figure S2. Morphology of U2OS cells after knocking down TRIM28.
After infecting U2OS cells with shRNA lenti virus with GFP for 36 hours, GFP fluorescence under fluorescence microscope and cell morphology under bright light were observed.
