# Supplementary material for: TRIM28 inhibits alternative lengthening of telomere phenotypes by protecting SETDB1 from degradation
Source: Cell Biosci. 2021 Jul 30;11:149. doi: 10.1186/s13578-021-00660-y (PMC8325274; doi:10.1186/s13578-021-00660-y)
Supplement: Supplementary file 3 — Additional file 3. TRIM28 deletion promotes telomere elongation. [file 13578_2021_660_MOESM3_ESM.pdf]

Additional File 3: Figure S3

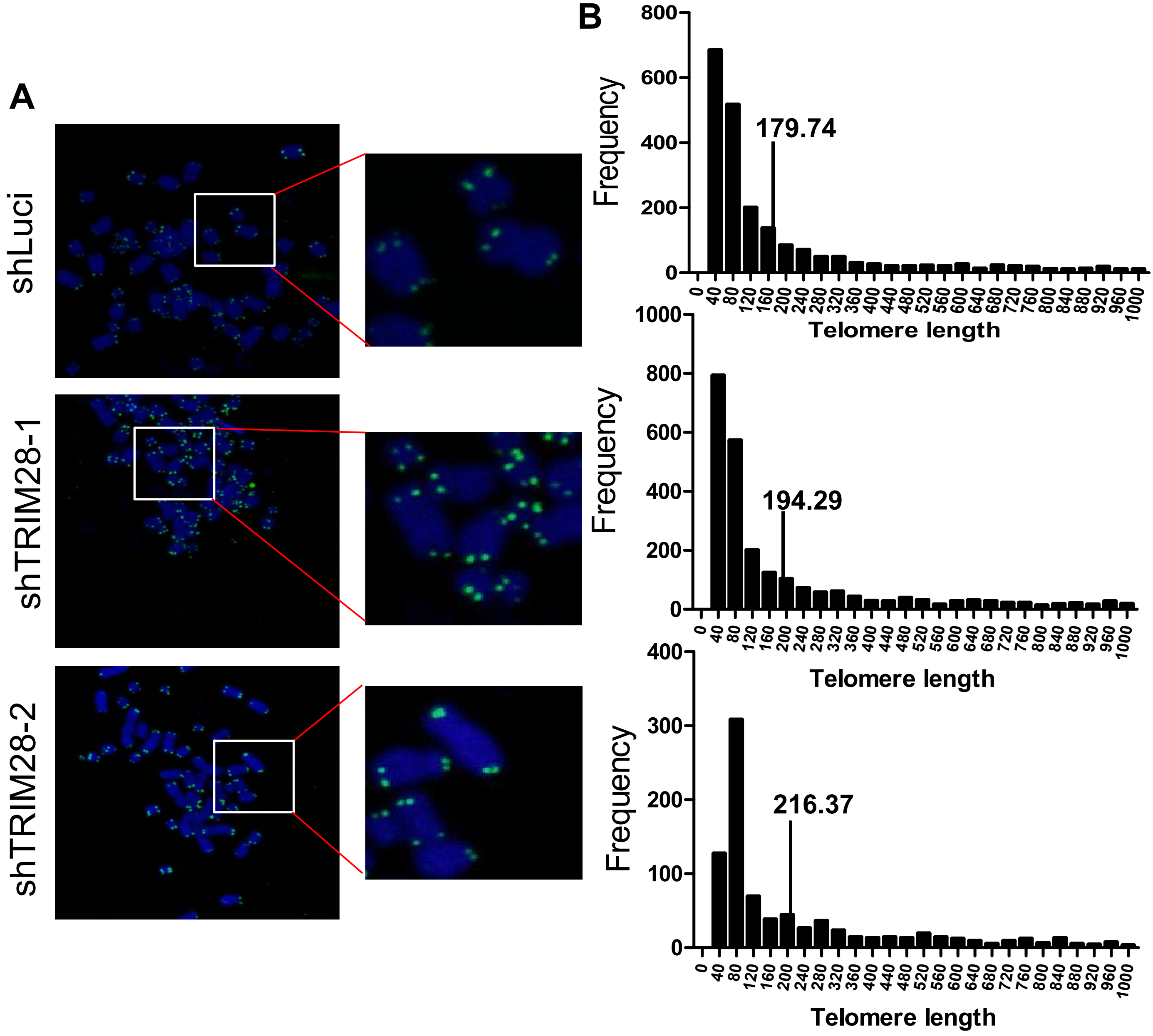

Figure S3. TRIM28 deletion promotes telomere elongation.
(A) Q-FISH analysis of TRIM28 KD U2OS cells was performed using a telomere probe (green). DAPI was used to stain chromosomes. (B) Data from (A) were quantified to assess relative telomere length
