# Supplementary material for: TRIM28 inhibits alternative lengthening of telomere phenotypes by protecting SETDB1 from degradation
Source: Cell Biosci. 2021 Jul 30;11:149. doi: 10.1186/s13578-021-00660-y (PMC8325274; doi:10.1186/s13578-021-00660-y)
Supplement: Supplementary file 4 — Additional file 4. U2OS cells of knocking down SETDB1 show morphology and decreased C-circle levels similar to the TRIM28-deficient cells. [file 13578_2021_660_MOESM4_ESM.pdf]

Additional File 4: Figure S4

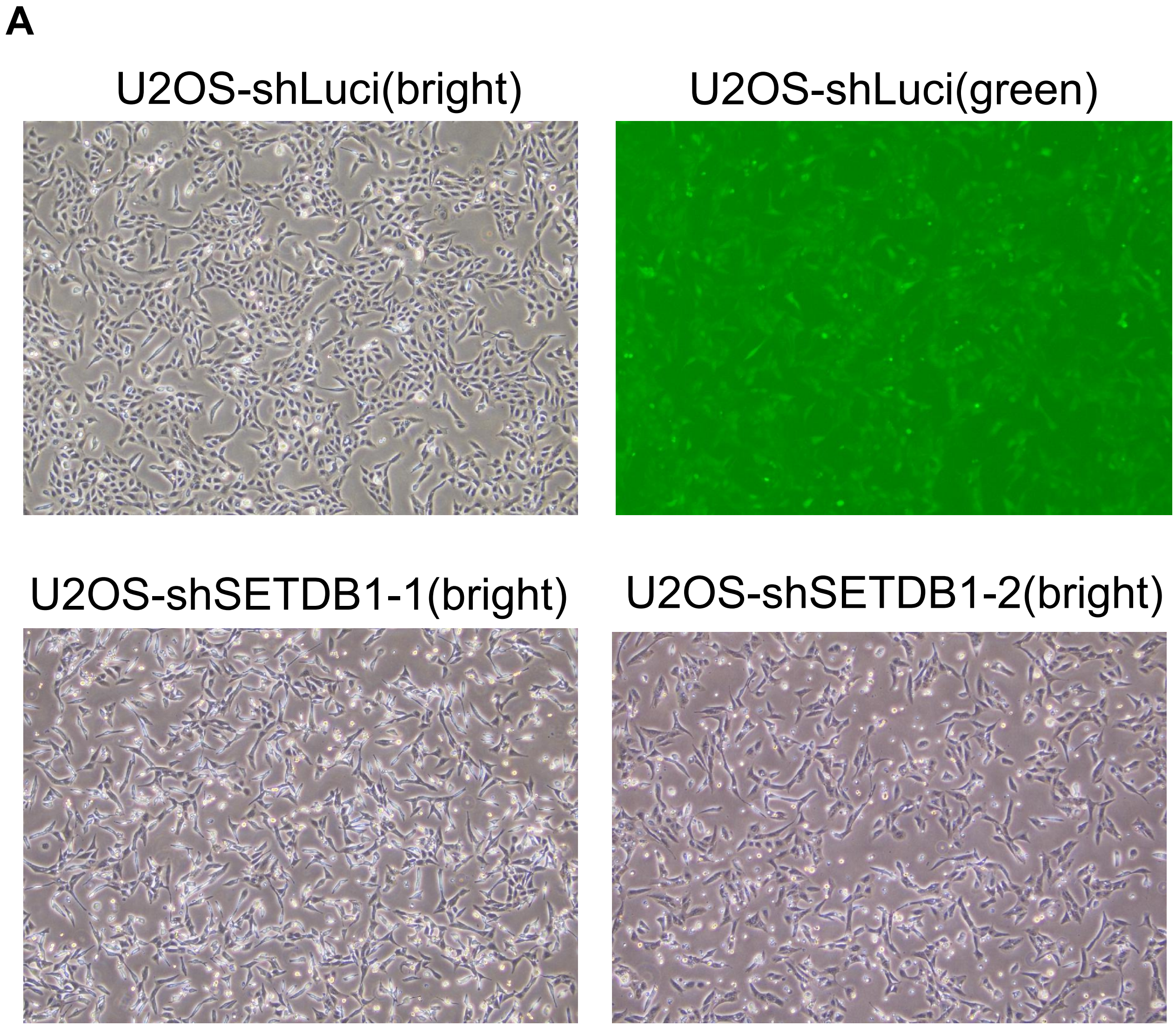

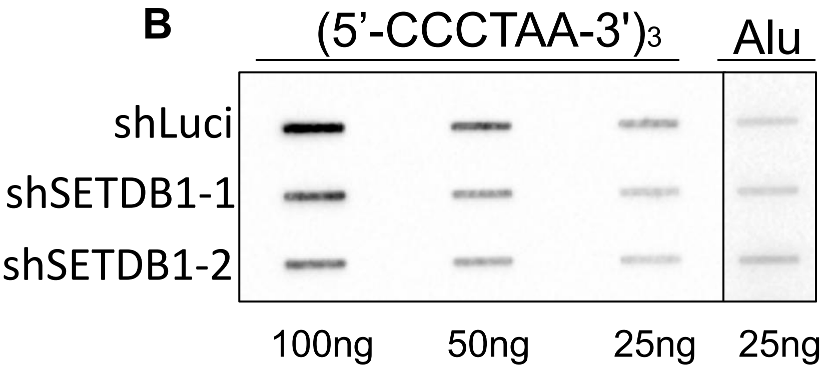

Figure S4. U2OS cells of knocking down SETDB1 show morphology and decreased C-circle levels similar to the TRIM28-deficient cells.
(A) Cell morphology after infecting U2OS cells with shLuci or shSETDB1 lenti virus with GFP for 36 hours. (B)U2OS genomic DNA (25, 50, or 100ng) from shLuci and shSETDB1 cells were used for the C-circle assay. An Alu repeat probe served as input control.
